# Severe pain at the end of life: a population-level observational study

**DOI:** 10.1186/s12904-020-00569-2

**Published:** 2020-04-30

**Authors:** A. Meaghen Hagarty, Shirley H. Bush, Robert Talarico, Julie Lapenskie, Peter Tanuseputro

**Affiliations:** 1 Department of Medicine, Division of Palliative Care, University of Ottawa, Ottawa, Ontario Canada; 2 Bruyère Research Institute, Ottawa, Ontario Canada; 3 Ottawa Hospital Research Institute, 1053 Carling Avenue, Ottawa, ON K1Y4E9 Canada; 4 ICES, Population Health and Primary Care, Ottawa, Ontario Canada

**Keywords:** Pain, End-of-life, Palliative care, Palliative medicine, Palliative homecare

## Abstract

**Background:**

Pain is a prevalent symptom at the end of life and negatively impacts quality of life. Despite this, little population level data exist that describe pain frequency and associated factors at the end of life. The purpose of this study was to explore the prevalence of clinically significant pain at the end of life and identify predictors of increased pain.

**Methods:**

Retrospective population-level cohort study of all decedents in Ontario, Canada, from April 1, 2011 to March 31, 2015 who received a home care assessment in the last 30 days of life (*n* = 20,349). Severe daily pain in the last 30 days of life using linked Ontario health administrative databases. Severe pain is defined using a validated pain scale combining pain frequency and intensity: daily pain of severe intensity.

**Results:**

Severe daily pain was reported in 17.2% of 20,349 decedents. Increased risk of severe daily pain was observed in decedents who were female, younger and functionally impaired. Those who were cognitively impaired had a lower risk of reporting pain. Disease trajectory impacted pain; those who died of a terminal illness (i.e. cancer) were more likely to experience pain than those with frailty (odds ratio 1.66).

**Conclusion:**

Pain is a common fear of those contemplating end of life, but severe pain is reported in less than 1 in 5 of our population in the last month of life. Certain subpopulations may be more likely to report severe pain at the end of life and may benefit from earlier palliative care referral and intervention.

## Background

Uncontrolled pain is consistently listed by patients as a primary source of fear for end-of-life care [1–3]. Palliative care aims to provide relief of pain and other physical symptoms in addition to supportive care for patients and their families at the end of life [4, 5]. Pain is often considered one of the more treatable symptoms in palliative care [6] and a request for assistance with pain management is a common reason for referral to palliative care physician specialists and palliative care teams. Uncontrolled pain is a common reason for palliative patients to present to acute care. Nearly one in ten emergency department visits from oncology patients in the last months of life cited pain as reason for visit [7]. Additionally, nearly 20% of patients who die in hospital experience some degree of pain [8]. Identification of those patients at risk for increased pain near the end of life is important for prompt initiation of a palliative approach and consideration of specialist palliative care referral [6, 9] as there is evidence that pain may be mitigated by palliative care intervention and home visits [10].

The bulk of the current data on the prevalence of pain is limited to specific populations. A systematic review examining studies between 1965 and 2006 demonstrated the pooled prevalence of pain in patients with advanced cancer was 64% [11]. Additionally, increased pain has been reported in advanced cancer patients with mental health illnesses, including depression and anxiety [12–14]. Estimates of the prevalence of pain in various late stage non-malignant populations [i.e., congestive heart failure (CHF), end-stage renal disease, chronic obstructive pulmonary disease (COPD)] range from 47 to 93% [15–17]. Studies of pain in persons with dementia have consistently demonstrated lower rates of reported pain [18, 19]. These studies, however, do not provide a sense of the prevalence of pain across the general population at end of life nor between disease trajectories (frailty, terminal illness, organ failure, sudden death). This is important as current evidence demonstrates disparities between disease trajectory and access to palliative care services [20]. An American retrospective observational study (*N* = 4703) demonstrated clinically significant pain in 47% of the population in the last month of life (as reported using non-validated 2 question measurement: participant “often troubled by moderate to severe pain”) [21]. The authors found pain was associated with proximity to death, arthritis and certain demographic factors such as sex, age, race and income. To our knowledge, no studies to date have captured in detail how pain varies across end-of-life trajectories, a wide variety of comorbid chronic diseases, home-based palliative care services, living arrangement (e.g., presence of a family caregiver) and other important patient characteristics such as impairment in function and cognition.

Our goal was to explore pain at the end of life across a wide variety of patient characteristics at a population level. To address the deficit in knowledge, we used multiple health linked databases providing access to detailed covariates in order to observe the frequency and severity of pain in the last month of life. We aimed to identify predictive or protective factors for pain at the end of life as well as potential risk factors that could be targeted for screening and prompt initiation of pain management strategies and palliative care referral.

## Methods

We conducted a population-based retrospective observational study using linked health administrative databases held at ICES. Our population included all decedents in Ontario, Canada from April 1, 2011 to March 31, 2015 (most recent, complete data available at time of analysis) who received a Resident Assessment Instrument–Home Care (RAI-HC) [22] assessment in the last 30 days of life. The RAI-HC database contains RAI-HC assessments which are conducted for all Ontarians seeking to receive long-stay home care (i.e., anticipated greater than 60 days). These assessments are conducted by trained assessors with input from the clinic team, the patient’s chart, the patient, and caregivers. Demographics, symptomatology, and detailed covariates were collected from each assessment. These covariates include: cognitive functioning, caregiver and living arrangements, activities of daily living (ADLs) on a 0–6 point performance scale (describing the discrete stages of loss in personal hygiene, toileting, locomotion and eating), instrumental activities of daily living (IADLs) (ordinary housework, meal preparation and phone use) [23]. Ethics approval was obtained from the Sunnybrook Health Sciences Centre Research Ethics Board in Toronto, Canada and from the Ottawa Health Science Network Research Ethics Board in Ottawa, Canada.

### Data sources

Encrypted health card numbers were used as unique identifiers and linked across several administrative databases held at ICES (Additional file 1). All data were de-identified and anonymized. Deaths and demographics including age and sex were captured from the Registered Persons Database (RPDB). Postal codes of residence were used to derive neighborhood income and rurality at the time of death through the Postal Code Conversion Files which are derived from the Statistics Canada 2011 census. The presence of chronic conditions at death was captured using previously developed—and in some cases validated— chronic disease databases held at ICES [24]. A total of 17 chronic diseases were examined and the number of diseases identified was totaled for each individual [25–31]. End-of-life trajectories (i.e., frailty, terminal illness, sudden death, organ failure, other) were captured using cause of death information from the Ontario Registrar General Database (ORGD) – deaths. The International Classification of Diseases (ICD-10) codes used to group deaths into these four categories, including validation in the Canadian population, are described elsewhere [20, 32–34].

Designated palliative homecare (e.g., from nurses, nurse practitioners, and personal support workers) and physician home visits were captured between 30 days to 6 months prior to death. Palliative home care was captured when a patient was given an end-of-life designation by home care services, which allows them to access additional and often specialized palliative care services. Physician home visits were identified using physician billing claims for services delivered at home, captured in the Ontario Health Insurance Plan (OHIP) database (Additional file 2). The subset of home visits delivered by palliative care physician specialists were identified using a validated definition of greater than 10% of all billings in the previous 2 years classified as palliative care [35]. Palliative home visits and services delivered by non-physician specialties (e.g. nurse practitioners, spiritual care, personal support workers, social workers, etc.) that occurred outside of designated publicly-funded palliative home care (i.e. out-pf-pocket expenses or private insurance) is not captured in available health administrative databases and were therefore not included in our analyses.

### Pain at end of life

Reported pain was captured using the RAI-HC database. Data was captured from those who received a RAI-HC assessment in the last month of life, the period associated with the highest pain scores [21]. A validated pain scale that combines pain intensity and frequency from the RAI-HC was applied to generate a four-point pain scale from no pain to severe pain occurring daily [36]. In this scale, severe daily pain was equivalent to an average of 5/10 on a visual analog scale. As pain beyond 4/10 has been shown to be associated with decreased functional status and quality of life [37, 38], we elected to compare decedents with severe daily pain to those without severe daily pain.

### Analysis

A logistic regression model was run for the primary outcome of severe daily pain in the last 30 days of life. Decedents with severe daily pain were compared to those without severe daily pain. Covariates of interest included demographics, comorbidities, functional status, and physician home visits in the 6 months to 1 month prior to death. Additionally, we examined the effect of a palliative care specialist being involved in at least one of the visits. The multivariable model examined the independent effect of potential predictors of pain that are available in health administrative databases: age, sex, neighborhood income quintile, rurality, functional status (i.e. ADLs and IADLs), Cognitive Performance Scale (CPS) [39] score, number of comorbidities, and end-of-life trajectories. All analyses were conducted using SAS 9.3 (SAS Institute Inc., Cary, NC).

## Results

In Ontario, between April 1, 2011 to March 31, 2015, there were 370,524 deaths. We captured data from 20,349 decedents who received a RAI-HC assessment in the last month of life (5.5% of total decedent population). The average age of our cohort was 81.4 years. The majority were female (51.6%) and lived in an urban setting. 42.8% had 5 or more chronic conditions. Less than 1 in 5 people (17.2%) reported severe daily pain using the validated pain scale (Table 1), with 30.3% of decedents reporting no pain. The majority (73.8%) felt they had adequate pain control at baseline or with medications, however 42.4% described pain that disrupted usual activities.

**Table 1:** Reported pain in decedents with a RAI-HC^a^ assessment in the last 30 days of life

	N	COL%
**Pain Frequency**		
No pain	6181	30.28
Less than daily	2036	9.97
Daily-one period	1262	6.18
Daily-multiple periods (e.g. morning and evening)	10,936	53.57
**Pain Intensity**		
No pain	6188	30.31
Mild	3211	15.73
Moderate	7419	36.34
Severe or excruciating	2776	13.6
Times when pain is horrible	821	4.02
**Pain disrupts usual activities**		
No	11,764	57.62
Yes	8651	42.38
**Pain - Adequate Medication**		
Yes/No pain	15,072	73.83
Medications do not adequately control pain	3407	16.69
Pain present, medication not taken	1936	9.48
**Pain Scale**		
No pain	6184	30.29
Less than daily pain	2036	9.97
Daily pain but not severe	8680	42.52
Severe daily pain	3515	17.22

^a^Resident Assessment Instrument–Home Care

### Factors associated with severe daily pain

#### Demographics

**Fig. 1:**
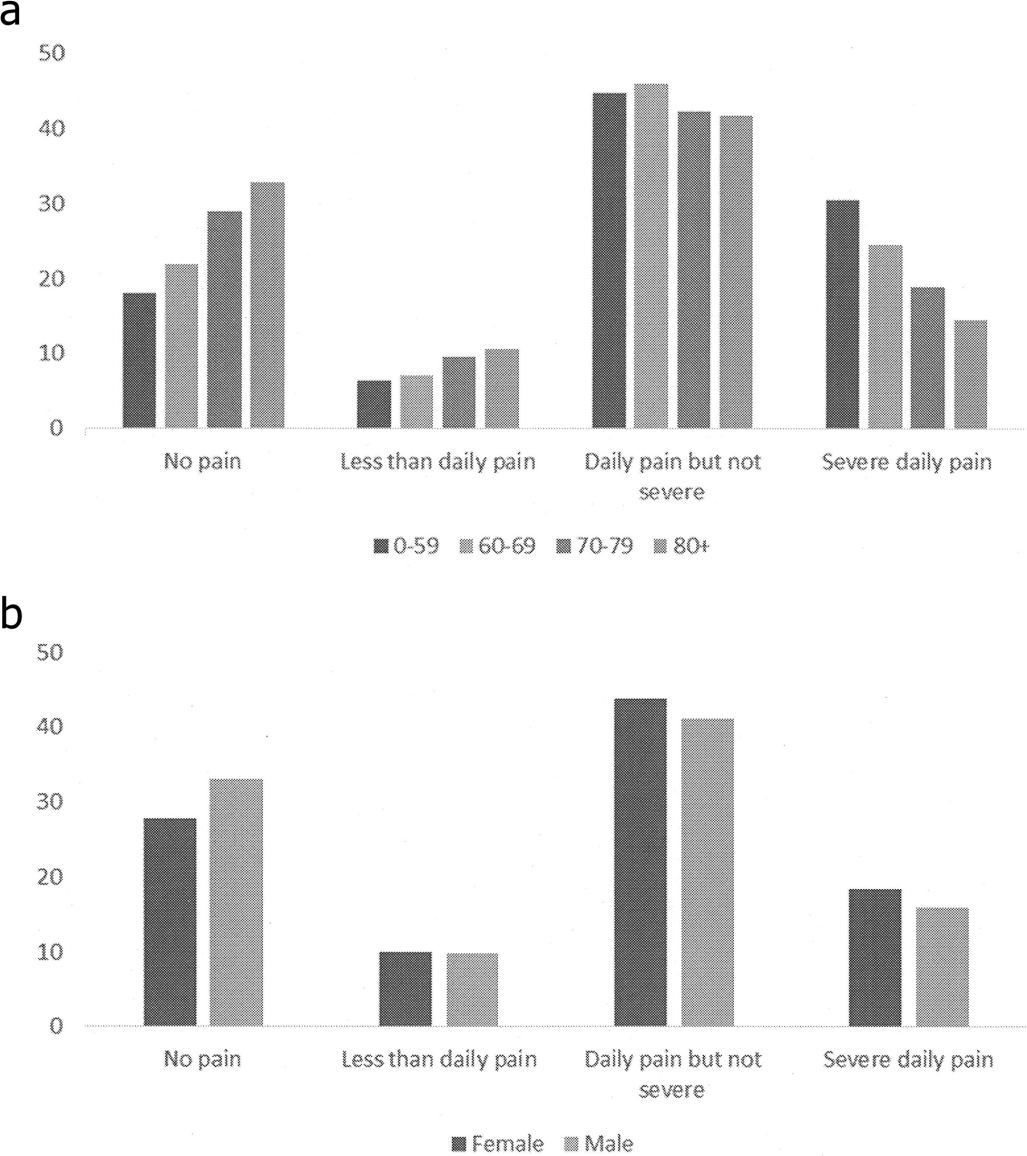
**a**. Pain scale percentages stratified by age in years. **b**. Resident Assessment Instrument-Home Care pain scale percentages stratified by sex

The proportion of severe daily pain was higher in those who died at a younger age (Fig. 1a).

Among female decedents, 18.4% reported severe daily pain compared to 15.9% of male decedents (Fig. 1b; Table 2). Younger decedents had a higher risk severe daily pain; 34.0% of 0–49-year-olds compared to only 13.3% of those aged 90+. Rurality and income were not found to significantly impact risk of severe daily pain. Those with 5+ chronic conditions reported more severe daily pain (17.8%) than those with 0–2 or 3–4 (17.5 and 16.3% respectively).

**Table 2:** Cohort characteristics by pain severity in the last 30 days of life

	No severe daily pain	(%)	Severe daily pain	(%)	All
	N		N		N
**Age**					
0–49	161	66.0%	83	34.0%	244
50–59	559	70.4%	235	29.6%	794
60–69	1452	75.4%	474	24.6%	1926
70–79	3285	81.0%	773	19.0%	4058
80–89	7181	84.7%	1297	15.3%	8478
90+	4206	86.7%	643	13.3%	4849
**Sex**					
Male	8281	84.1%	1569	15.9%	9850
Female	8563	81.6%	1936	18.4%	10,499
**Income Quintile**					
Highest	2990	83.8%	576	16.2%	3566
High	3141	82.4%	673	17.6%	3814
Middle	3307	82.6%	695	17.4%	4002
Low	3679	83.2%	744	16.8%	4423
Lowest	3727	82.0%	817	18.0%	4544
**Rurality**					
Urban	13,807	82.9%	2850	17.1%	16,657
Rural	3037	82.3%	655	17.7%	3692
**Palliative Home Care**					
No	13,205	84.1%	2488	15.9%	15,693
Yes	3639	78.2%	1017	21.8%	4656
**Physician Home Visit**					
No	14,711	83.0%	3008	17.0%	17,719
Yes - Non-PC^a^ specialist	1817	81.8%	405	18.2%	2222
Yes - PC specialist	372	78.5%	102	21.5%	474
**Number of Chronic Conditions**					
0–2	3744	82.5%	795	17.5%	4539
3–4	5938	83.7%	1157	16.3%	7095
5+	7162	82.2%	1553	17.8%	8715
**Cancer (any)**					
No	11,968	83.6%	2341	16.4%	14,309
Yes	4876	80.7%	1164	19.3%	6040
**Dementia**					
No	13,355	81.2%	3092	18.8%	16,447
Yes	3489	89.4%	413	10.6%	3902
**Diabetes Mellitus**					
No	10,571	83.1%	2145	16.9%	12,716
Yes	6273	82.2%	1360	17.8%	7633
**Mental Health (other)**					
No	15,816	82.9%	3268	17.1%	19,084
Yes	1028	81.3%	237	18.7%	1265
**Mood and Anxiety Disorders**					
No	14,460	83.2%	2926	16.8%	17,386
Yes	2384	80.5%	579	19.5%	2963
**Osteo-arthritis**					
No	7842	85.0%	1384	15.0%	9226
Yes	9002	80.9%	2121	19.1%	11,123
**Renal Failure**					
No	13,855	83.3%	2787	16.7%	16,642
Yes	2989	80.6%	718	19.4%	3707
**Rheumatoid Arthritis**					
No	16,066	83.1%	3261	16.9%	19,327
Yes	778	76.1%	244	23.9%	1022
**Stroke**					
No	14,962	82.6%	3146	17.4%	18,108
Yes	1882	84.0%	359	16.0%	2241

^a^Palliative Care

Reported severe daily pain varied with living arrangements (Table 3): decedents who lived in a private community home with or without homecare reported higher severe daily pain (17.5, 18.2%) than those who lived in an assisted living or residential care facility (15.9, 14.5%). Those who lived with relatives were more likely to report severe daily pain (with spouse:18.4%, with spouse and others:19.0%, with child:18.7%) compared to those who lived alone (17.1%) or with non-relatives (15.3%). Decedents with reported caregiver stress had increased pain compared to those with no caregiver stress (18.3% vs. 16.4%).

**Table 3:** Cohort characteristics by pain severity in the last 30 days of life

	No severe daily pain	(%)	Severe daily pain	(%)	All
	N		N		N
**ADLS**^**a**^					
Independent	3180	83.2%	641	16.8%	3821
Supervision required	1475	82.4%	316	17.6%	1791
Limited impairment	3113	83.1%	633	16.9%	3746
Extensive assistance required (I)	1900	83.2%	383	16.8%	2283
Extensive assistance required (II)	3017	83.4%	602	16.6%	3619
Dependent	2760	80.5%	667	19.5%	3427
Total dependence	1399	84.2%	263	15.8%	1662
**IADLs**^**b**^					
No difficulty in any of three IADLs	97	93.3%	7	6.7%	104
Some difficulty in one IADL but no difficulty in the other two	158	88.3%	21	11.7%	179
Some difficulty in two IADLs but no difficulty in the other one	474	85.3%	82	14.7%	556
Some difficulty in all three IADLs	94	89.5%	11	10.5%	105
Great difficulty in one IADL but less than great difficulty in the other two	1240	82.0%	273	18.0%	1513
Great difficulty in two IADLs but less than great difficulty in the other one	7373	79.9%	1856	20.1%	9229
Great difficulty in all three IADLs	7408	85.5%	1255	14.5%	8663
**Cognitive Performance Scale (CPS)**					
Intact	3230	79.7%	824	20.3%	4054
Borderline intact	2260	79.2%	595	20.8%	2855
Mild impairment	5853	82.1%	1275	17.9%	7128
Moderate impairment	2395	86.3%	381	13.7%	2776
Moderate/severe impairment	722	88.4%	95	11.6%	817
Severe impairment	1352	88.1%	183	11.9%	1535
Very severe impairment	1032	87.2%	152	12.8%	1184
**Caregiver Stress**					
Yes	7383	81.7%	1652	18.3%	9035
No	9461	83.6%	1853	16.4%	11,314
**Where Lived at Time of Referral**					
Missing	8659	83.2%	1747	16.8%	10,406
Private home/apt. With no home care services	5184	81.8%	1156	18.2%	6340
Private home/apt. With home care services	1803	82.5%	383	17.5%	2186
Board and care/assisted living/group home	768	84.1%	145	15.9%	913
Residential care facility	241	85.5%	41	14.5%	282
Other	189	85.1%	33	14.9%	222
**Who Lived with at Time of Referral**					
Missing	8659	83.2%	1747	16.8%	10,406
Lived alone	2300	82.9%	476	17.1%	2776
Lived with spouse only	2798	81.6%	633	18.4%	3431
Lived with spouse and other(s)	666	81.0%	156	19.0%	822
Lived with child (not spouse)	1105	81.3%	254.0	18.7%	1359
Lived with other(s) (not spouse or children)	572	84.5%	105	15.5%	677
Lived in group setting with non-relative(s)	744	84.7%	134	15.3%	878
**Disease Trajectory**^**c**^					
Frailty	3317	87.3%	481	12.7%	3798
Organ Failure	7596	85.0%	1344	15.0%	8940
Sudden Death	671	83.4%	134	16.6%	805
Undetermined	323	83.0%	66	17.0%	389
Other	531	79.5%	137	20.5%	668
Terminal Illness	4406	76.6%	1343	23.4%	5749

^a^Activities of Daily Living

Extensive assistance—Client performed part of activity on own (50% or more of subtasks), but help of following type(s) were provided 3 or more times:

(I) Weight-bearing support—OR—

(II) Full performance by another during part (but not all) of last 3 days

Dependent—Client involved and completed less than 50% of subtasks on own (includes 2+ person assist), received weight bearing help

Total dependence—Full performance of activity by another

^b^Instrumental Activities of Daily Living

^c^Disease trajectories - frailty (e.g., dementia), organ failure (e.g., congestive heart failure), terminal illness (e.g., cancer)

#### Functional status

In examining ADLs (Table 3), reported severe daily pain was highest in those who were dependent (19.5%) and lowest in those who were totally dependent (15.8%). Similarly, pain severity generally trended up with increasing impairment in IADLs to a maximum of great difficulty in 2 out of 3 IADLs as collected on the RAI-HC (20.1%). Those decedents with great difficulty carrying out all three IADLs reported lower than average severe daily pain (14.7%).

#### Clinical factors

Reported severe daily pain decreased with worsening cognitive impairment, with 20.3% of cognitively intact persons reporting severe daily pain compared to 12.8% with very severe cognitive impairment. Pain scores varied with end-of-life trajectory. Those with frailty (e.g., dementia), organ failure (e.g., COPD or CHF) and sudden death had a lower proportion reporting severe daily pain than those with terminal illness (e.g., cancer) (Table 3). The following chronic conditions were associated with increased risk of severe daily pain (Table 2): rheumatoid arthritis (23.9%), mood and anxiety disorders (19.5%), renal failure (19.4%), cancer (19.3%), osteoarthritis (19.1%) and other mental health illness (18.7). Many cardiac conditions (acute myocardial infarction, congestive heart failure, hypertension) as well as chronic neurological conditions [history of stroke (16.0%) and dementia (10.6%)] were associated with lower than average reports of severe daily pain.

Physical symptoms as reported on the RAI-HC associated with higher severe daily pain include dyspnea (19.2%), anorexia (22.2%), emesis (29.5%), constipation (31.4%) and edema (20.2%) (Table 4). Increasing severity of pressure ulcers were also associated with higher rates of pain. Additionally, psychological symptoms such as loneliness and sad mood were associated with increased reports of severe daily pain.

**Table 4:** Symptomology self-reported in RAI-HC^a^ by pain severity in the last 30 days of life

	Severe Daily Pain				All
	No		Yes		
	N	%	N	%	N
**Shortness of Breath**					
No	9029	84.6	1643	15.4	10,672
Yes	7815	80.8	1862	19.2	9677
**Loss of Appetite**					
No	11,202	86.0	1825	14.0	13,027
Yes	5642	77.1	1680	22.9	7322
**Vomiting**					
No	16,126	83.5	3197	16.5	19,323
Yes	718	70.5	301	29.5	1019
**Constipation**					
No	16,271	83.4	3243	16.6	19,514
Yes	573	68.6	262	31.4	835
**Delusions**					
No	16,359	82.8	3400	17.2	19,759
Yes	485	82.2	105	17.8	590
**Hallucinations**					
No	15,925	82.9	3282	17.1	19,207
Yes	919	80.5	223	19.5	1142
**Sad Mood**^b^					
0	12,052	85.9	1981	14.1	14,033
1	2692	79.6	691	20.4	3383
2	2100	71.6	833	28.4	2933
**Pressure Ulcer**^c^					
0	13,824	83.6	2718	16.4	16,542
1	1595	81.7	357	18.3	1952
2	1066	79.1	282	20.9	1348
3	254	73.8	90	26.2	344
4	105	64.4	58	35.6	163
**Edema**					
No	10,689	84.6	1943	15.4	12,632
Yes	6155	79.8	1562	20.2	7717
**Loneliness**					
Unknown	4879	85.2	845	14.8	5724
No	10,826	82.5	2303	17.5	13,129
Yes	1139	76.1	357	23.9	1496
**Client Felt/Was Advised to Reduce Drinking**					
No	16,573	82.8	3446	17.2	20,019
Yes	271	82.1	59	17.9	330
**Compliance/Adherence With Medications**					
Always Compliant	14,905	83.0	3059	17.0	17,964
Compliant > 80%	1427	79.7	364	20.3	1791
Compliant < 80%	355	82.9	73	17.1	428
No Medications	157	94.6	9	5.4	166
**Time Since Last Hospital Stay**					
Missing	8659	83.2	1747	16.8	10,406
In hospital	2923	85.2	509	14.8	3432
> 180 days	1626	80.1	404	19.9	2030
Within last week	1045	81.8	232	18.2	1277
Within 8–14 days	920	84.7	166	15.3	1086
Within 15–30 days	827	82.1	180	17.9	1007
More than 30 days	844	76.0	267	24.0	1111

^a^Resident Assessment Instrument–Home Care

^b^Sad Mood- 0. Indicator not exhibited in last 3 days, 1. Exhibited 1–2 of last 3 days 2. Exhibited on each of last 3 days

^c^Presence of an ulcer anywhere on the body. Ulcers include any area of persistent skin redness (Stage 1); partial loss of skin layers (Stage 2); deep craters in the skin (Stage 3); breaks in skin exposing muscle or bone (Stage 4). [Code 0 if no ulcer, otherwise record the highest ulcer stage (Stage 1–4)

#### System factors

A minority of decedents received designated palliative home care or a physician home visit between 30 days to 6 months prior to death, at 22.9 and 13.2% respectively. Decedents who received designated palliative home care had higher severe daily pain in the last 30 days of life than those without (21.8% vs 15.9%). A trend was also demonstrated toward increased pain in those who received a physician home visit. Pain trended upward with time since self-reported admission to hospital with 14.8% of those in hospital versus 19.9% in those who had not reported a hospitalization in the previous 180 days.

### Logistic regression models for odds of severe daily pain

Adjusting for multiple covariates as listed in our methods, females had greater odds of having severe daily pain [OR = 1.25; 95% Confidence Interval (CI): 1.16 to 1.35] (Table 5). The odds ratio of severe daily pain was 0.31 in the decedents aged 90+ compared to 0–49 (95% CI: 0.23 to 0.42). Those with severe or very severe cognitive impairment had an OR of 0.68 and 0.52, respectively, compared to those who were cognitively intact. When examining disease trajectory, compared to frailty, those with terminal illness were more likely to report severe daily pain (OR 1.66, (95% CI: 1.46 to 1.88). Decedents with designated palliative home care had greater odds of increased pain compared to those without [OR 1.13 (95% CI: 1.03 to 1.24)]. Conversely, the trend seen with physician home visits was no longer statistically significant for specialist or non-specialist home visits when all covariates were accounted for [OR 1.12 (95% CI: 0.99 to 1.26) and 1.14 (95% CI: 0.91 to 1.44)].

**Table 5:** ﻿Multivariate logistic regression for factors associated with severe daily pain among the last 30 days of life

Effect	Odds Ratio Estimate	Lower 95% Confidence Limit for Odds Ratio	Upper 95% Confidence Limit for Odds Ratio
**Age**			
0–49	ref	ref	ref
50–59	0.79	0.58	1.08
60–69	0.60	0.45	0.80
70–79	0.44	0.33	0.59
80–89	0.36	0.27	0.47
90+	0.31	0.23	0.42
**Sex**			
Male	ref	ref	ref
Female	1.25	1.16	1.35
**Income Quintile**			
Highest	ref	ref	ref
High	1.10	0.97	1.24
Middle	1.07	0.94	1.21
Low	1.03	0.92	1.17
Lowest	1.08	0.95	1.21
**Rurality**			
Urban	ref	ref	ref
Rural	0.98	0.89	1.08
**ADLs**^**a**^			
Independent	ref	ref	ref
Limited impairment	1.12	0.98	1.28
Supervision required	1.10	0.94	1.29
Extensive assistance required (I)	1.26	1.08	1.46
Extensive assistance required (II)	1.31	1.13	1.51
Dependent	1.76	1.53	2.04
Total dependence	2.05	1.63	2.59
**IADLs**^**b**^			
No difficulty in any of three IADLs	ref	ref	ref
Some difficulty in one IADL only	2.04	0.83	5.03
Some difficulty in two IADLs only	2.69	1.20	6.04
Some difficulty in all three IADLs	2.16	0.80	5.87
Great difficulty in one IADL but less than great difficulty in the other two	3.57	1.63	7.83
Great difficulty in two IADLs but less than great difficulty in the other one	3.90	1.79	8.51
Great difficulty in all three IADLs	3.09	1.41	6.77
**Palliative Home Care**			
No	ref	ref	ref
Yes	1.13	1.03	1.24
**Physician Home Visit**			
No Physician Home Visit	ref	ref	ref
Physician Home Visit Non Specialist	1.12	0.99	1.26
Palliative Care Specialist	1.14	0.91	1.44
**Cognitive Performance Scale (CPS)**			
Intact	ref	ref	ref
Borderline intact	1.10	0.97	1.24
Mild impairment	0.97	0.88	1.08
Moderate impairment	0.75	0.65	0.87
Moderate/severe impairment	0.61	0.48	0.78
Severe impairment	0.68	0.56	0.82
Very severe impairment	0.52	0.40	0.68
**Number of Chronic Conditions**			
0–2	ref	ref	ref
3–4	1.09	0.98	1.21
5+	1.34	1.21	1.49
**Trajectory**			
Frailty	ref	ref	ref
Organ Failure	1.06	0.94	1.19
Sudden Death	1.28	1.04	1.58
Undetermined	1.26	0.95	1.68
Other	1.59	1.28	1.97
Terminal Illness	1.66	1.46	1.88

^a^Activities of daily living

^b^Instrumental activities of daily living

## Discussion

We examined the proportion of severe daily pain reported in the last 30 days of life using population-based administrative databases. We observed that less than 1 in 5 decedents (17.2%) report severe daily pain. This level of pain is considered inadequately treated and would likely be associated with lower quality of life and functional impairment [37, 38]. We identified multiple demographic, clinical and system factors associated with increased end-of-life pain, many of which have not been previously described. Notably, disease trajectory impacted reported severe daily pain at the end of life. Those with terminal illness (i.e. cancer) and other had higher odds of reporting pain than those with frailty, sudden death or organ failure (cardiac or pulmonary). Interestingly, renal failure is categorized into the other disease trajectory and was associated with increased reported pain. Although this is a condition that is not typically considered inherently painful, it is possible that pain in this population may be undertreated, possibly due to fear of using analgesic medications that may worsen renal function or are renally cleared. Additionally, increased pain reported by females and younger decedents could be hypothesized to be related to the specific illness or trajectory related to these populations; however, this trend is persistent when disease trajectory was accounted for. The increased reported pain in those receiving palliative services may have been related to referral bias where those with increased pain are more likely to receive a palliative care referral. However, only a small minority received a palliative home care designation or physician home visit despite being close to death. This is consistent with other jurisdictions signaling large room for improvement in access to palliative care services [35, 40].

Our study addresses a gap in the previous literature by examining end-of-life pain in a large sample, using a validated pain scale and conducting analyses adjusting for multiple potential confounders. The proportion of pain reported in this study is lower than previously reported by other population research [21]. This may be attributed to our study examining those with daily severe pain compared to previous research including intensity (moderate-severe) but not considering frequency when determining clinical significance. Previous studies [11–13, 21] have demonstrated an association between pain and select comorbidities: arthritis, cancers and mental health conditions, which was again shown in our population. We demonstrated lower reported pain in persons with neurological impairment (dementia and post-stroke). Decreased reported pain in those with reduced cognitive functioning was maintained with confounders such as age, frailty and gender accounted for. This is consistent with previous studies demonstrating that pain may be underreported in those with cognitive impairment [18, 19]. It is difficult to infer if perceived pain levels are in fact lower or if those with cognitive impairment are unable to vocalize pain.

### Strengths and limitations

We examined a wide array of health care services at the end of life for a large, population-based decedent cohort. This is possible in Ontario, comprising of approximately 40% of the Canadian population, where well-developed health administrative databases are linked at an individual level for a range of publicly-funded health services. Previous studies have focused on specific populations or had limited access to other health care services utilized by decedents. We recognize the data used for this study is relatively old, although there were no significant policy or practice changes since 2015 that would reasonably be expected to influence the relevance of our findings to current practice. While used widely as a clinical assessment tool in many settings, we also acknowledge that the validation for the RAI-HC pain scale was completed in elderly patients in nursing homes, potentially limiting the generalizability of this scale. Additionally, one of our primary limitations is that our data is collected from those who have received a RAI-HC assessment in the last month of life. This may limit the generalizability to those in long-term care home (nursing home), community, or hospital settings who have not been assessed for publicly funded home services (about 40% of decedent population) [41]. This approach also does not capture palliative home care received through private (out-of-pocket) expenses or nurse practitioner palliative home visits. Nevertheless, the RAI-HC provided us with a rare large population-based cohort that contained detailed information about patient-centered variables and outcomes (symptoms, living arrangements, caregiver information), beyond what has previously been presented in literature.

## Conclusion

We observed multiple demographic, clinical and system factors associated with increased pain at the end of life. Clinicians should recognize severe daily pain is common but perhaps not proportional to the fear of suffering in pain that many experience when contemplating end of life [2]. Regardless this is still a significant number of people who report severe pain, and prompt screening and management of pain should be considered, particularly for those with increased risk factors. Improvements in access and quality of care likely would reduce the prevalence of severe pain at the end of life, given previous studies showing large gaps in palliative care provision [41].

## Supplementary information

**Additional file 1.** Databases held at ICES used in this study. Includes database name and a description of the type of data (variables) obtained from each database.

**Additional file 2.** Definitions of Palliative Home Care and Palliative Physician Home Visits. Includes a list and description of billing (physician) and service (home care) codes used to determine if a patient received either service.

## Data Availability

The data that support the findings of this study are available from ICES, but restrictions apply to the availability of these data according to ICES policies and provincial and federal privacy laws to protect individual patient data, and so are not publicly available. As the data custodian, all requests for data should go through ICES. Please contact the corresponding author (PT) should you have questions about accessing study data.

## Abbreviations

CHF: Congestive heart failure
COPD: Chronic obstructive pulmonary disease
RAI-HC: Resident assessment instrument – home care
ADLs: Activities of daily living
IADLs: Instrumental activities of daily living
RPDB: Registered Persons Database
ORGD: Ontario Registrar General Database
ICD-10: International classification of diseases
OHIP: Ontario health insurance plan
CPS: Cognitive performance scale
OR: Odds ratio
CI: Confidence interval
