# Supplementary material for: Severe pain at the end of life: a population-level observational study
Source: BMC Palliat Care. 2020 Apr 30;19:60. doi: 10.1186/s12904-020-00569-2 (PMC7193354; doi:10.1186/s12904-020-00569-2)
Supplement: Supplementary file 1 — Additional file 1. Databases held at ICES used in this study. Includes database name and a description of the type of data (variables) obtained from each database. [file 12904_2020_569_MOESM1_ESM.docx]

**Additional File 1**

Databases held at the ICES used in this study:

| Database | Description |
| --- | --- |
| Home Care Database (HCD)  Resident Assessment Instrument-Home Care (RAI-HC) | Data from the Ontario Association of Community Care Access Centers, responsible for providing all publicly funded home care |
| National Rehabilitation Reporting System (NRS) | Data from participating adult inpatient rehabilitation facilities and programs across Ontario |
| CIHI-DAD | Administrative, clinical, and demographic data on all hospital discharges in Ontario |
| National Ambulatory Care Reporting System (NACRS) | Captures all emergency department visits in Ontario |
| NACRS | Select outpatient visits held in hospitals, including dialysis clinics and cancer care clinics |
| Ontario Health Insurance Plan (OHIP) Claims Database | Claims data for physicians in Ontario – includes claims in both inpatient and outpatient settings. |
| OHIP | Health professionals for provincially insured services, such as select midwives, oral surgeons, chiropractors, optometrists, and physiotherapists. Some care may occur for inpatients |
| OHIP | Outpatient laboratory services. Does not include laboratory services for inpatients |
| Ontario Drug Benefit (ODB), Assistive Devices Program (ADP) | Drugs for those over 65 years, on social assistance, residents of LTC, home care recipients, Trillium drug program and special drugs program recipients for those qualifying for assistance. Select medically-necessary devices including home oxygen and respiratory devices. |
| Registered Persons Database (RPDB) | Demographic data from the provincial health insurance plan |
| Ontario Registrar General Database (ORGD) | Vital statistics records for deaths, which includes causes of death. |
