# Supplementary material for: Severe pain at the end of life: a population-level observational study
Source: BMC Palliat Care. 2020 Apr 30;19:60. doi: 10.1186/s12904-020-00569-2 (PMC7193354; doi:10.1186/s12904-020-00569-2)
Supplement: Supplementary file 2 — Additional file 2. Definitions of Palliative Home Care and Palliative Physician Home Visits. Includes a list and description of billing (physician) and service (home care) codes used to determine if a patient received either service. [file 12904_2020_569_MOESM2_ESM.docx]

**Additional File 2**

Definition of Palliative Homecare: Service Recipient Codes 95 or 54

Home based physician visits were captured in the Ontario Health Insurance Plan (OHIP) billing database through the following codes:

- An assessment by a physician, rendered in a patient’s home that satisfies, at a minimum, all of the requirements of an intermediate assessment - A901
- Travel billing codes for visits to patient’s home for any reason - B960, B961, B962, B963, B964
- Travel billing code for palliative care home visit - B966
- First person seen billing premium for visits to patient’s home for any reason - B990, B992, B993, B994, B996
- First person seen billing premium for palliative care home visit - B998
